# New-age vaccine adjuvants, their development, and future perspective

**DOI:** 10.3389/fimmu.2023.1043109

**Published:** 2023-02-24

**Authors:** Shailendra Kumar Verma, Pooja Mahajan, Nikhlesh K. Singh, Ankit Gupta, Rupesh Aggarwal, Rino Rappuoli, Atul Kumar Johri

**Affiliations:** ^1^ School of Biotechnology, Jawaharlal Nehru University, New Delhi, India; ^2^ School of Life Sciences, Jawaharlal Nehru University, New Delhi, India; ^3^ Integrative Biosciences Center, Department of Ophthalmology, Visual and Anatomical Sciences, Wayne State University, School of Medicine, Detroit, MI, United States; ^4^ Microbiology Division, Defence Research and Development Establishment, Gwalior, India; ^5^ Glaxo Smith Kline (GSK) Vaccine, Siena, Italy

**Keywords:** improved vaccines, adjuvants, mucosal vaccine, immune response, infectious disease

## Abstract

In the present scenario, immunization is of utmost importance as it keeps us safe and protects us from infectious agents. Despite the great success in the field of vaccinology, there is a need to not only develop safe and ideal vaccines to fight deadly infections but also improve the quality of existing vaccines in terms of partial or inconsistent protection. Generally, subunit vaccines are known to be safe in nature, but they are mostly found to be incapable of generating the optimum immune response. Hence, there is a great possibility of improving the potential of a vaccine in formulation with novel adjuvants, which can effectively impart superior immunity. The vaccine(s) in formulation with novel adjuvants may also be helpful in fighting pathogens of high antigenic diversity. However, due to the limitations of safety and toxicity, very few human-compatible adjuvants have been approved. In this review, we mainly focus on the need for new and improved vaccines; the definition of and the need for adjuvants; the characteristics and mechanisms of human-compatible adjuvants; the current status of vaccine adjuvants, mucosal vaccine adjuvants, and adjuvants in clinical development; and future directions.

## Introduction

The invention of vaccines has been considered as one of the triumphs of medical research. Immunization not only stops the spread of infection during childhood but also provides a lifetime of protection against some diseases. However, the scientific community continues to face challenges in developing ideal vaccines against many infectious diseases, i.e., plague, tuberculosis, malaria, human immunodeficiency virus (HIV), and severe acute respiratory syndrome coronavirus 2 (SARS-CoV-2), due to immunological barriers such as inadequate immune response and weak immunological memory against vaccines (1–3). Apart from these obstacles, vaccine safety issues such as adverse effects in a population suffering from rare genetic disorders, systemic and local reactogenicity caused by diphtheria and tetanus toxoids along with whole cell pertussis (DTwP), and waning immunity shown by diphtheria and tetanus toxoids along with acellular pertussis (DTaP) (4) have been considered unacceptable, which further increases the impact of the challenge to solve the vaccine problem for emerging or reemerging disease threats. These challenges warrant new strategies that can help to understand immune responses for immunization and introduce new ways to induce robust immunity without sacrificing quality and safety (5).

The worldwide scientific community has recently witnessed significant disease outbreaks, i.e., SARS in 2003, the H1N1 influenza pandemic of 2009, Ebola virus in 2014 (6), the plague in Madagascar in 2017 (7), the Nipah outbreak in India in 2018 (8), and, the most notable thus far, the ongoing COVID-19 pandemic (9). In 2014, the Ebola epidemic created huge panic in developed countries as the mortality rate was found to be quite high in West African countries (10). These incidences force the scientific community not only to be alert but also to open up new avenues in pursuing new strategies to elucidate the mechanistic approach to develop an effective vaccine against these emerging pathogens (11). At present, the ongoing COVID-19 pandemic has greatly affected human lives worldwide and devastated the global economy; therefore, the scientific community at large is busy developing an effective and safe vaccine against SARS-CoV-2 (12).

Currently, inactivated, live-attenuated, subunit, and nucleic acid-based vaccines are the four types of vaccines available for the human population (13, 14). Live-attenuated vaccines comprise the whole pathogen that can replicate in the host body and induce strong immune responses. Live-attenuated vaccines have been observed to be the most effective against polio, Measles, Mumps and Rubella (MMR), chicken pox, influenza, rotavirus, and yellow fever. Killed whole-pathogen vaccines are inactivated by heat or chemicals [inactivated polio (Salk) and hepatitis A], are noninfectious, and are mostly safe. Inactivated (killed) vaccines have been observed to induce weak and short-term immunity, thus the need for boosters to achieve complete protection (15). It has been found that DTwP from India, which is a kind of licensed inactivated vaccine, bypasses waning immunity and shows long-term protective efficacy (4, 16).

Similar to inactivated whole-pathogen vaccines, purified or recombinant subunit vaccines do not contain live components of the pathogen, but they consist only of the antigenic parts of the pathogen, which makes them different from the former. Subunit vaccine antigens have been poorly immunogenic, hence the need to add components to enhance their protective immunity. Subunit vaccines sometimes use epitopes that are shown to identify and interact with T cells or immunoglobulins. Subunit vaccines have been generally documented safe in terms of toxicity and reactogenicity, as subunit vaccines comprise purified or recombinant antigens rather than the whole cell (17). There are a few very successful examples of subunit vaccines, such as hepatitis B virus (HBV), influenza virus (injection), and pertussis vaccines. The developed subunit vaccines have been found to be poorly immunogenic, and thus, multiple boosters and suitable adjuvantation are necessary to augment their protective potential. Recently, mRNA vaccines (nucleic acid-based vaccines) have been found to play an important role during the COVID-19 pandemic. These mRNA-based vaccines provide good immune response by directing the production of antibodies, thus preventing serious complications (18).

## The need for adjuvants

Adjuvants were first discovered in 1920 by Gaston Ramon, a French scientist who observed in his findings that the inclusion of aluminum salts to vaccines enhanced their potential. The term “adjuvant” originated from the Latin word *adjuvare*, which means “to help.” Adjuvants are generally not immunogenic, but they modulate the immune responses in formulation with the given vaccines, thus not only reducing the required dose of vaccine but also extending immune memory. Typically, the vaccines are formulated with suitable adjuvants to augment the immune responses to the administered vaccine antigen to evaluate the potential to halt the contagion. Another important role of adjuvants is to direct humoral and cell-mediated immune responses to generate pathogen-specific immunity (19–22).

**Figure 1:**
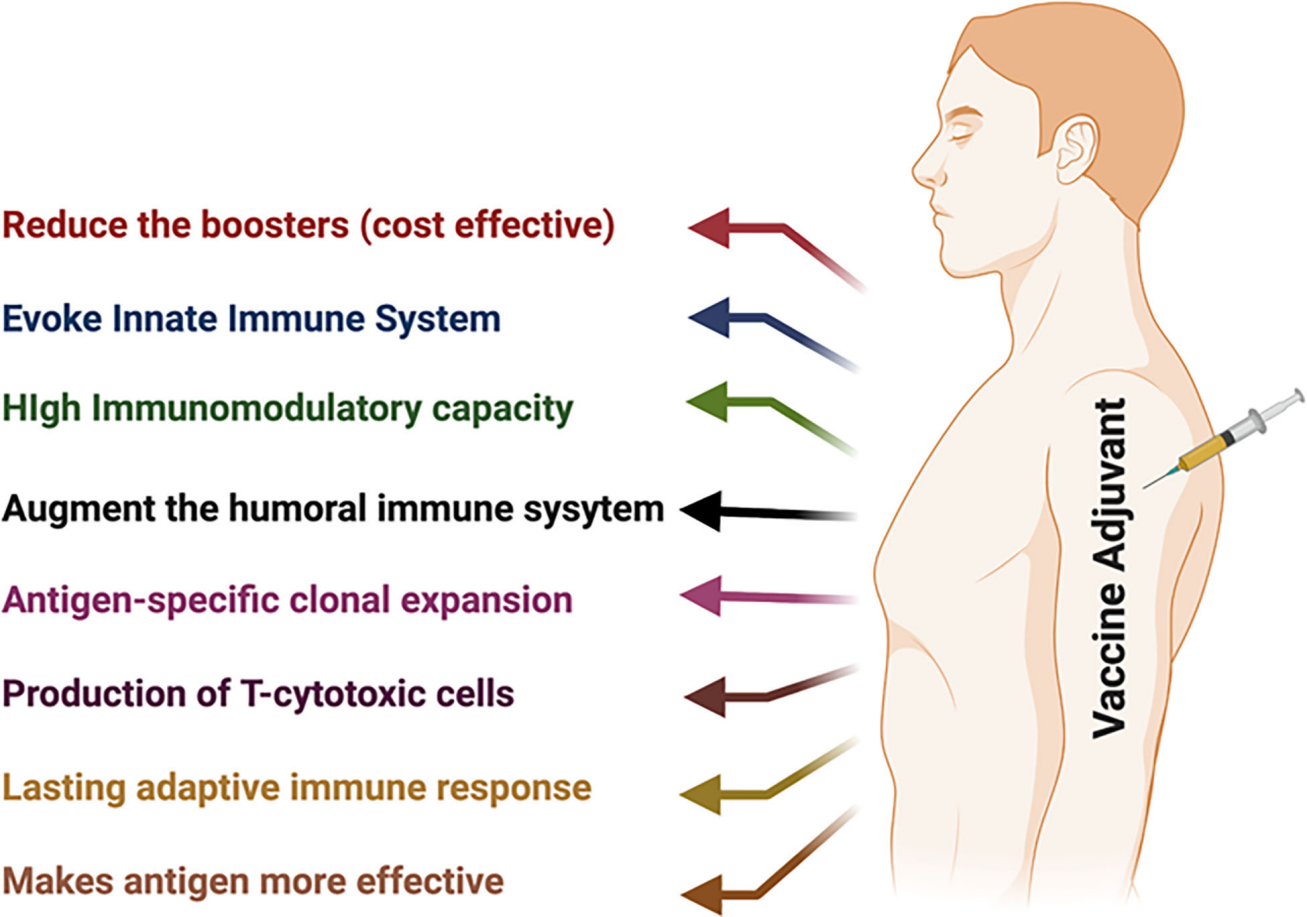
Schematic representation of vaccine adjuvants and their benefits.

At present, adjuvants are exploited (a) to augment the immune response to the given vaccine and enhance the antibody response and the number of recipients that were vaccinated; (b) to enhance rates of seroconversion in individuals with diminished responsiveness due to age, illness, or therapeutic interventions, e.g., the use of the adjuvant MF59 with the influenza vaccine to improve/increase the response in aged individuals (23, 24); and (c) to reduce the dose and the number of boosters of vaccine antigens (25–27) as the ability of an adjuvant to allow comparable responses with considerably lower amounts of vaccine antigen might be crucial in regions where vaccine production facilities are limited and immunization is urgent in public in general. The demand for vaccines with multiple boosters poses noteworthy challenges worldwide. Adjuvants can decrease the number of required boosters to provide complete protection (26–28) (**Figure 1**).

Another reason for formulating a vaccine with an adjuvant is to attain qualitative modulation of the immune response. Adjuvants are used to a greater extent for underdeveloped vaccines to modulate types of immune responses that are not effectively stimulated by vaccine antigens without adjuvants. Adjuvants are used in preclinical and clinical studies (i) to provide functionally suitable immune responses (e.g., humoral or cellular, Th1, Th2, and Th17); furthermore, it has been observed that balanced Th1/Th2/Th17 responses increase the duration of T-cell responses and prolong mouse survival (29–33); (ii) to enhance long-term memory cells (e.g., T-cell memory) (34–36); (iii) to provide the initial rapid response that can be essential in a pandemic (37–39); and (iv) to modify the breadth, specificity, or affinity of the response (39, 40) (**Figure 1**). In this review, our main objective is to focus on the modulation of the immune response using various adjuvants.

## Characteristics and mechanisms of action

### Aluminum salts (alum)

Aluminum salts have been clinically approved, and they are the most widely used adjuvants in human vaccines. These adjuvants are made up of the precipitates of aluminum phosphate and aluminum hydroxide for the adsorption of vaccine antigens. Brenntag Biosector, Chemtrade, and SPI Pharma™ are some of the manufacturers that prepare these formulations. Alhydrogel^®^, Rehydragel™, and Adju-Phos^®^ are tradenames of alum that are available in the market (41). Aluminum salts as adjuvants have been utilized for more than 80 years in vaccine research and usually stimulate the Th2 type of immune response (42, 43). Alum has been approved as a component of licensed human vaccines, i.e., *Haemophilus influenzae* type b (Hib), both hepatitis A and B viruses, tetanus, meningococcal virus, human papilloma virus (HPV), diphtheria, and the most recent SARS-CoV-2 (11, 41, 44–47). The mechanism of action of alum is almost known, and it is now clear that depot formation is not an essential step for the activity of alum as an adjuvant (48–50). Alum as an adjuvant primarily evokes innate immunity (50–52). Alum stimulates B-cell differentiation to augment antibody production (53). It is also well documented that alum stimulates the Th2 type of immune response in mice, but in humans, almost all the vaccine antigens in formulation with the alum adjuvant induce a mixed type of response, i.e., Th1 and Th2 (54, 55). Alum triggers the NLRP3 inflammasome to express interleukin (IL)-1β after *in vitro* priming of macrophages and dendritic cells (DCs) with lipopolysaccharide (LPS) (56). However, *in vivo*, the adjuvanticity of alum does not support the data (50, 51). It has been observed that alum stimulates the Th2 type of immune response and produces IL-4, IL-5, IgG1, and IgE (43, 57). Another functional activity performed by alum is the initiation of a signaling cascade by using DCs to carry out actin-mediated phagocytosis that leads to the activation of two kinase proteins (Src and Syk), which, in turn, mobilizes Ca^2+^ and finally activates the transcription factor NFAT (calcineurin-nuclear factor of activated T cells), resulting in the production of IL-2 (33, 58–60). In addition, alum mediates its adjuvanticity by activating the cascade of complement proteins (41, 61–63). Alum as an adjuvant is highly advantageous due to its safety, vaccine antigen stabilization, and the modulation of high-production and long-lasting antibody titers. Vaccines that have been formulated with an alum adjuvant cannot be filter sterilized, lyophilized, or frozen (64).

### Adjuvants in emulsion forms (oil-in-water)

#### MF59 and AS03 adjuvants

**Figure 2:**
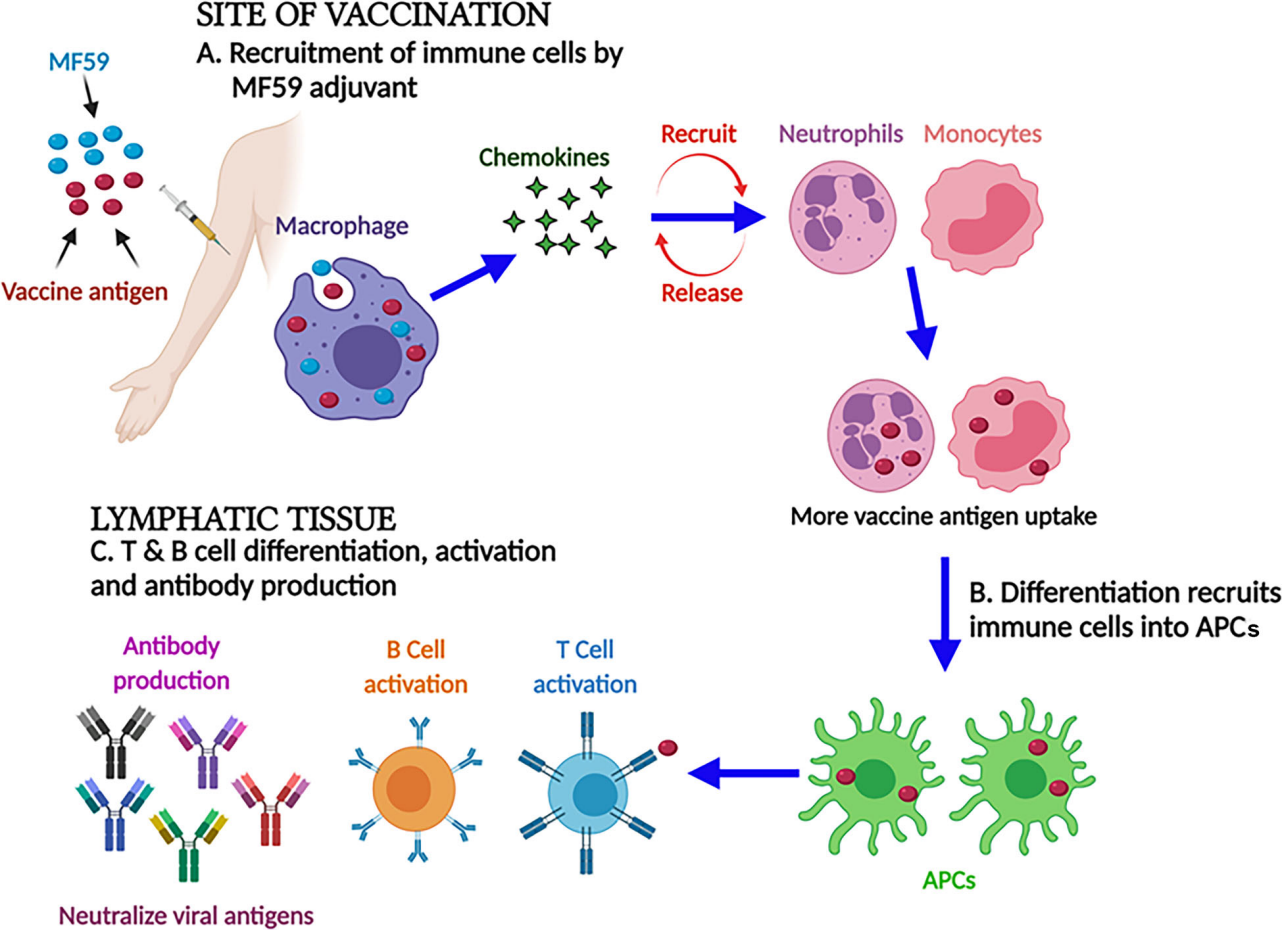
MF59 adjuvant and its mechanism of action. At the injection site, MF59 adjuvant-activated macrophages secrete the chemokines that stimulate and recruit the immune cells. Differentiation converts immune cells into antigen-presenting cells to activate B and T cells to impart strong humoral and cellular immune responses.

MF59 is a highly safe and effective oil-in-water emulsion of squalene oil. Unlike Freund’s adjuvants, squalene is more readily metabolized and highly purified for vaccine development. Recently, MF59 has been licensed by Fluad™ (Seqirus, Melbourne, Australia) as an important component of flu vaccine for old people, and it has also been successfully used in infants and children later on (65, 66). Apart from this, it was also licensed to be used as a pandemic vaccine against H1N1 in children, infants, and pregnant women (67). It is evident from the findings that the MF59 adjuvant trivalent inactivated influenza vaccine (TIV) elicited a strong humoral and cellular immune response in infants in comparison to non-MF59 adjuvanted influenza vaccines (68, 69). The formulation of MF59 significantly modulated the weak efficacy of the influenza vaccine in infants. Subsequently, the HBV vaccine in formulation with the MF59 adjuvant was observed to induce a 100 times stronger immune response in comparison to alum (70). Similar to other adjuvants, the mechanism of action of MF59 is not yet fully elucidated. The effectiveness of the MF59 adjuvant does not depend on the formation of a depot at the site of vaccination because of the short half-life of MF59 (49, 71). However, the MF59 adjuvant has shown the potential to elicit a strong IgG and cell-mediated immune response (72). Moreover, MF59 can induce monocytes, macrophages, and DCs to express and secrete chemokines, i.e., CCL4, CCL2, CCL5, and CXCL8, which recruit more leukocytes for the reuptake of more vaccine antigens. Finally, this differentiation converts immune cells into antigen-presenting cells (APCs). Furthermore, these APCs migrate to lymph nodes where they induce an adaptive immune response as shown in **Figure 2** (67, 73, 74).

AS03 is also an oil-in-water emulsion adjuvant that includes squalene, α-tocopherol, and polysorbate 80 (75). The inclusion of α-tocopherol into the AS03 formulation made it different from other oil-in-water emulsion adjuvants (76). AS03 was first used in formulation with a vaccine against malaria (77). Recently, this adjuvant has been used for human vaccination against influenza. Recent clinical trials have shown that the AS03 adjuvant with the influenza vaccine elicited a strong immune response (78). In addition, the AS03 adjuvant vaccine was found to induce a robust immune response in infants as well (79). Furthermore, AS03 evokes immunity by stimulating Nuclear factor kappa B (NF-κB), a proinflammatory cytokine, and chemokines. Later, it recruits immune cells such as monocytes and macrophages. The advantage of applying this adjuvant in formulation with the pandemic vaccine is its capability of generating a strong humoral immune response with a lower dose of vaccine antigen, i.e., 3.75 or 7.5 μg per strain in comparison to 15 µg per strain in conventional trivalent inactivated influenza vaccines. Since 2009, ~4.7 million doses of AS03-adjuvanted A(H1N1) vaccines have been injected in children (80, 81).

#### Army liposome formulation

This formulation was developed by the U.S. military, made up of cholesterol and liposomes containing saturated phospholipids and monophosphoryl lipid A (MPLA) (82). There are improved versions of army liposome formulation (ALF), i.e., ALF adsorbed to aluminum hydroxide (ALFA), ALF containing the QS21 saponin (ALFQ), and ALFQ adsorbed to aluminum hydroxide (ALFQA) (83, 84). It has been found that the vaccine candidate circumsporozite protein of *Plasmodium falciparum* adjuvanted with ALFA imparted adequate humoral and cellular immune responses (85). The WRAIR (Walter Reed Army Institute of Research) malaria vaccine branch developed and tested the protective efficacy of FMP013 (falciparum malaria protein-013) and FMP014 (a self-assembling protein nanoparticle) SAPN (two new synthetic malarial antigens) with an ALFQ adjuvant (86–88).

Recombinant gp120 adjuvanted with ALFA induced cross-reactive antibodies against different subtypes of HIV-1 (89). As regards ALF and ALFQ in formulation with the recombinant HIV-1 envelope gp140 protein, ALF induces a dominant Th2 type of immune response, while ALFQ induces a more balanced Th1 and Th2 type of immunity (90, 91). In addition, the ALFQ adjuvant activates innate immune responses, upregulates APOBEC3 (apolipoprotein B mRNA-editing enzyme catalytic polypeptide-like family), an anti-HIV protein, and maintains a proinflammatory environment, as a result of which, MDMs (monocyte-derived macrophages) that are permissive to HIV-1 infection become capable of restricting HIV-1 infection (92).

#### Virosomes (lipids and glycoproteins)

Virosomes display the attributes of an adjuvant system and are known for their biodegradable and nontoxic qualities. Generally, virosomes do not generate anti-virosome antibodies (93). Virosomes are small spherical unilamellar lipid membrane vesicles (150 nm) embedded with viral envelope proteins, such as neuraminidase and hemagglutinin of the influenza virus. These proteins are integrated into phosphatidylcholine bilayer liposomes. These prepared virosomes are devoid of nucleocapsid including the genetic material of the source virus (94). These proteins facilitate virosome membranes to bind with cell receptors, mediating pH-dependent fusion with immune cells. Consequently, virosomes transport their contents, i.e., vaccine antigen(s), directly to their target cells, evoking an antigen-specific immune response yet carrying a weak immunogenic antigen (95). Virosomes are virus-like particles that allow the presentation of vaccine antigen to both major histocompatibility complex (MHC) class I and class II to elicit both B-cell and T-cell immune responses (95–99).

The types of immune response evoked by the virosome adjuvant system depend on whether the antigenic epitopes are outside or inside the virosome. There are few established examples such as PeviPRO™, which induces a humoral immune response (27). The antigen is degraded in endosomes of the cell and, hence, mainly generates an MHC II antigen presentation. PeviTER™ formulated antigens not only elicit a CD4+ and CD8+ T-cell immune response but also generate a strong cytotoxic T lymphocyte (CTL) response. Virosome adjuvant system encapsulation presents vaccine antigens *via* the MHC I route because the antigen is delivered naturally into the APC cytosol (95). The approved vaccines such as Epaxal^®^ (for hepatitis A) and Inflexal^®^V (for influenza) (100) have successfully proven the excellence of virosomes. Thus far, these hepatitis A (Epaxal^®^) and influenza (Inflexal^®^V) vaccines have been authorized to be used in more than 45 countries, and more than 10 million people have been immunized to date. This new generation of vaccines offers additional benefits because they are effective even in immunosuppressed patients and in infants (101). Furthermore, they have a high safety profile as embedded viruses do not replicate. To the best of our knowledge, virosomes are no longer licensed to be used in humans.

#### AS04 (alum-adsorbed TLR-4 agonist)

To develop the novel adjuvant systems, aluminum salts have been used, mainly consisting of different Toll-like receptor (TLR) agonists absorbed on alum. Adjuvant system 04 (AS04) has already been approved for use in formulation with HPV (Cervarix) and HBV (Fendrix) vaccines. This adjuvant system includes alum in formulation with LPS that mainly constitutes 3-O-desacyl-4′-monophosphoryl lipid A (MPLA) from *Salmonella minnesota.* It is documented that MPLA retains the ability to stimulate innate immunity by interaction with TLR-4 (54). It further leads to induce NF-κB signaling and produce pro-inflammatory cytokines and chemokines that recruit the immune cells at the vaccination site and draining lymph nodes. An increase in the number of monocytes and DCs has been observed within a few hours of vaccination where they interact to stimulate antigen-specific T and B cells for strong cellular and humoral immune responses. There is no synergistic effect shown by alum with MPLA; however, a comparative study of MPLA and AS04 revealed that alum extends the cellular responses evoked by MPLA at the vaccination site. Hence, research findings suggest that the AS04 adjuvant induces innate immune responses by stimulating TLR-4 (54). The AS04-adjuvanted HBV vaccine elicits innate immune responses in humans (102). An elevated level of IL-6 and C-reactive protein was observed in the AS04-adjuvanted HBV immunized serum in comparison to the alum-adjuvanted HBV immunized serum. Yet, higher HBs Ag-specific T cells and antibodies were reported than those induced by the HBV vaccine in formulation with alum (103). Both HBV and HPV vaccines in formulation with AS04 elicit stronger humoral immune responses in comparison to the same vaccines when formulated with the alum adjuvant, signifying the importance of the TLR4 agonist MPLA adjuvant for human use (103–105).

#### RC-529 adjuvant

Sequential acid and base hydrolysis of LPS generates an MPLA that is known to retain various immunostimulatory functions of LPS. *In vitro* studies have shown that MPLA activates and stimulates the maturation of DCs and upregulates the human leukocyte antigen‐DR, CD80, CD86, CD40, and CD83 (106). MPLA is also known to induce the expression of Th1 and Th2 cytokines (106, 107) and augment the antigen-specific Tc cell response (108, 109). MPLA is an approved adjuvant and has been used in hepatitis B vaccine formulations. After this, synthetic mimetics such as aminoalkylglucosaminide 4-phosphates (AGPs) were characterized, and it was observed that they activate innate cells such as macrophages, DCs, B cells, and APCs. One such AGP compound, RC-529, was observed to activate the signal *via* TLR-4 and upregulate the costimulatory molecules on the cell surface including cytokines and chemokines (107). RC-529 was found to be an effective adjuvant in a clinical trial in which healthy volunteers were administered with a vaccine formulation against hepatitis B. In comparison to alum, RC-529 induced a significantly high production of antibodies in subjects who were administered with the vaccine formulation (108, 110). MPLA’s synthetic mimetic RC-529 was observed as safe and effective in clinical trials (111).

## Mucosal vaccine adjuvants

These adjuvants evoke the innate immune system of the host and help to provide protection against pathogens. Whole-cell-based vaccines using live-attenuated or killed pathogens usually consist of endogenous adjuvants, such as the products of the bacterial cell wall and their genomic DNA/RNA. These adjuvants act as pathogen-associated molecular patterns (PAMPs) and are adequate to stimulate adaptive immunity. However, subunit vaccines usually miss, or are unable to induce, an innate immune response, and thus, the inclusion of an adjuvant is essential to deliver successful vaccines (112). Most of the pathogens enter the host *via* either the intranasal or the oral route. Therefore, an effective mucosal vaccine is of utmost importance to prevent such kinds of mucosal transmitted diseases. The mucosal vaccine, in comparison to the intramuscular/subunit vaccine, provides noteworthy benefits such as low cost, noninvasiveness, and, most importantly, very low risk of transmission of blood-borne infections, especially to young children. However, due to the lack of ideal mucosal adjuvants, only a few mucosal vaccines are approved for human use (113).

Generally, mucosal adjuvants have two roles: first, they act as delivery vehicles, and second, they act as immunostimulatory molecules. However, some mucosal adjuvants exhibit the characteristics of both immunostimulators and delivery systems, such as chitosan and its derivatives (114). It is documented that mucosal adjuvants can stimulate protective local and systemic immunity that is crucial for successful mucosal immunizations against various infectious diseases (115–118). In addition, mucosal adjuvants perform many vital roles to provide protection against infections at distant as well as local sites. For example, Cytosine-phosphorothioate-guanine (CpG) oligodeoxynucleotides act as effective mucosal adjuvants for vaccinations *via* the nasal route against pathogens transmitted by blood transfusion and sexual activities (119, 120). Moreover, mucosal adjuvants are strong stimulators of immunity against tumors (121, 122). Among many mucosal adjuvants, the agonists of TLR and mutant enterotoxins are the two most appealing types because they are not only effective but also comparatively safe (115). So far, bacterially derived Adenosine diphosphate (ADP)-ribosylating enterotoxins are the most characterized mucosal adjuvants. This class of adjuvant comprises *Escherichia coli* heat-labile enterotoxin (LT), cholera toxin (CT), and mutants/subunits of LT and CT. These toxins stimulate an antigen-specific cellular and humoral immune response that includes CTLs, Th1, Th2, and Th17. Most importantly, these adjuvants stimulate antigen-specific IgA antibodies and long-lasting memory cells to vaccine antigens when administered *via* the mucosal route (123).

## How do heat-labile toxin/double-mutant heat-labile toxin mucosal adjuvants work?

The mucosal adjuvant LT is a polymeric protein of 84 kDa. This adjuvant retains an active form as AB5 that contains an A subunit and a pentameric B subunit. dmLT is a mutated form of its parent molecule LT (124, 125). The adjuvant dmLT immunomodulates the systemic as well as mucosal immune responses specific to the vaccine antigen after vaccination *via* the mucosal or parental route. It can simply be formulated with vaccine antigen(s) in an aqueous buffer. Due to the dual approach of the adjuvant, i.e., immunostimulatory characteristics and universal cell binding, the cell uptake of vaccine antigen(s) are many-fold high, and that is how the mucosal immunity is enhanced. This is the most suitable approach to deliver vaccine formulations, particularly for subunit vaccines to unapproachable sites, and specifically for sublingual (s.l.), oral (p.o.), and transcutaneous (t.c.i.) delivery. These strategies not only are needle-free but also reveal the capacity to enhance ease of administration and compliance. Moreover, it reduces the risk of transmission of the diseases from vaccinations using risky injections (126–129). The dmLT adjuvant evokes a strong Th17 response specific to the vaccine antigen and induces IL-17, and it provides protection against infections mainly in mucosal sites (130). The expression of IL-17 helps to increase the transport of secretory sIgA antibodies into the lumen of mucosal tissue by inducing B-cell differentiation into IgA-secreting cells (131–134). The dmLT adjuvant also stimulates the mucosal immune responses after parenteral vaccination (135–139). However, these studies have been tested only in animal models, and the use of this adjuvant in humans is yet to be determined.

LT, CT, and their related mutants have been very well characterized in the recent past (139–141). The subunits of the active form AB5 of LT or CT adjuvant function uniquely. Subunit B binds to the receptor and leads the entry into the cell. During mucosal vaccination, the B subunit helps in transporting the vaccine antigens throughout the mucosal sites (142). Subunit A is responsible for binding to ADP-ribosylation factors (ARFs), and ADP ribosylates Gsα, which results in an accumulation of Cyclic adenosine monophosphate (cAMP). Then, LT stimulates the activation of DCs, the expression of cytokines, and the stimulation of Th17 response (142, 143). It is also documented that the use of subunit A of LT alone evokes a mixed Th1/Th2/Th17 type of immune response but has a weaker impact than the active AB5 form of LT. The use of subunit B evokes a Th2/T regulatory cell (Treg)-skewed response (144). However, toxicity has always been a concern for these adjuvants.

**Figure 3:**
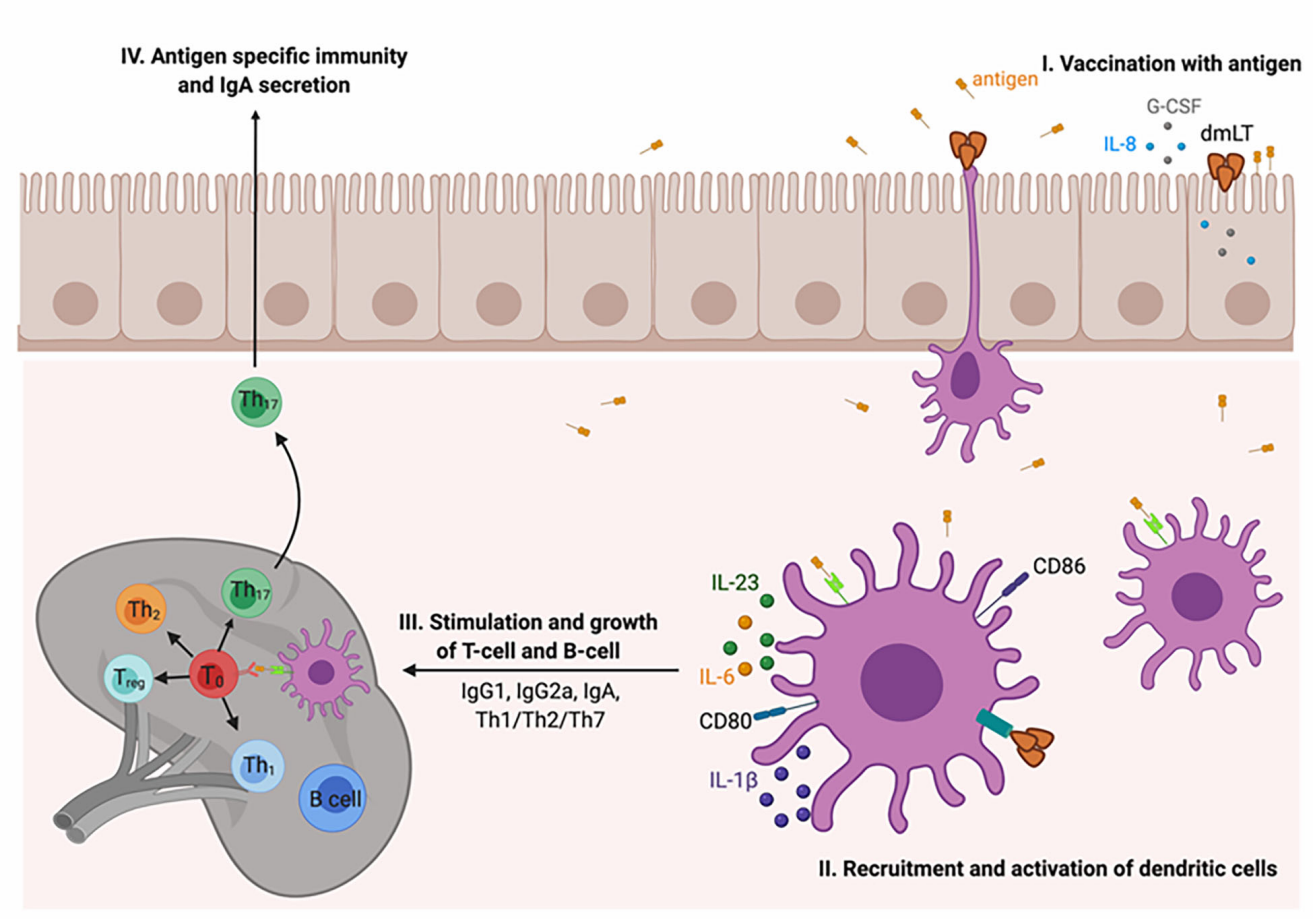
Mechanism of action of the dmLT adjuvant. (I) At the vaccination site, the dmLT adjuvant stimulates the innate immune response and activated epithelial cells secrete IL-8 and G-CSF. (II) Dendritic cells (DCs) are activated and recruited at the injection site where they process and present the antigen. It also upregulates the costimulatory molecules, i.e., CD80 and CD86, and stimulates the expression of IL-1, IL-6, IL-23, and G-CSF. (III) The activated DCs migrate to secondary lymphoid organs where they stimulate the differentiation of antibody-secreting plasma B cells and the generation of antigen-specific Th cells. Overall, a strong Th17 immune response is imparted. Th17 cells are identified as essential in immunity and in stimulating germinal center formation in secondary lymphoid organs as well as in enhancing IgA secretion.

How does the dmLT adjuvant induce the immune system? **Figure 3** depicts the immunologic cascade. (I) At the vaccination site, after the uptake of the vaccine antigen, the activation of the innate immune system takes place. The epithelial cells secrete cytokines and chemokines such as IL-8 and granulocyte colony-stimulating factor (G-CSF). (II) The DCs are recruited to the vaccination site and activated for antigen processing and presentation to the major histocompatibility complex (Ag-MHC). The upregulation of CD80 and CD86 and the secretion of polarizing cytokines, e.g., IL-1, IL-23, IL-6, and G-CSF, take place. (III) The activated DCs carrying the vaccine antigen then migrate to the secondary lymphoid organs and facilitate the differentiation of antigen-specific T helper and B cells into plasma cells secreting IgA and IgG antibodies. Finally, a mixed Th1/Th2/Th17 type of immune response is imparted with specifically strong stimulation of Th17 cells (124, 145–147) and mucosal homing markers (135). It has been documented that Th17 cells are an essential part of immunity for promoting germinal center formation in secondary lymphoid organs and for augmenting IgA antibody secretion (132–134).

The stimulated DCs by an LT/dmLT adjuvant induced a very high Th17 type of immune response due to the activation of caspase 1 inflammasome and, later, the expression of chemokines and cytokines such as IL-1 and IL-23. A scientific study reported that murine DCs activated with LT induced the expression of IL-1β, which is essential for the production of Th17 cells (148). Re-stimulation of human Peripheral blood mononuclear cells (PBMC) of immunized individuals with vaccine antigens in formulation with dmLT augmented the expression of IL-17A and IL-13 (146). In summary, a defined series of events takes place after immunization with a vaccine antigen in formulation with dmLT, which is an effective stimulator of APCs and vaccine immunity (123).

## TLR agonists as a mucosal vaccine adjuvant

Vaccines in formulation with TLR ligand-based adjuvants trigger and activate an innate immune response that helps in the augmentation of a protective potential imparted by the vaccine candidate. TLR-based adjuvants require an additional adaptor protein, MyD88, which stimulates APCs and B cells for antibody production. In addition, MyD88 signaling also induces germinal center formation that is imperative to produce antibody-secreting cells (149). TLR ligand-based adjuvants include the TLR9-dependent adjuvant CpG DNA (CpG) and the TLR2-dependent adjuvant Porin B (PorB), both of which require MyD88 for proper stimulation of APCs. CpG is an unmethylated bacterial DNA motif while PorB is an outer-membrane protein of *Neisseria meningitides*. It is documented that TLRs are expressed on APCs, including macrophages, DCs, and B cells. Therefore, the main purpose of using a TLR agonist in vaccine preparation as an adjuvant is to stimulate these cells to impart a robust immune response, linking innate and adaptive immunity (149). Various ligands for TLR4 have been not only used in preclinical studies but also approved by the Food and Drug Administration for clinical trials like Bacillus Calmette–Guerin (BCG) and MPLA. LPS, a well-known ligand for TLR4, was used as an inducer of acute inflammation *in vivo* for tumor regression in a mouse model (150). It has been reported that the TLR4 agonist, a second-generation lipid adjuvant like SLA-SE, promotes an increment in mucosal antibodies elicited by intramuscular immunization with an enterotoxigenic *E. coli* (ETEC) vaccine antigen in a mouse model (151). To test the efficacy of L-pampo, a combination of TLR1/2 and TLR3 agonists for the SARS-CoV-2 ferret model was utilized, which elicited a vigorous humoral and cell-mediated immune response (152). More recently, an orally administered SARS-CoV-2 vaccine (VXA-CoV2-1), which is currently in Phase III clinical trial, used the double-stranded RNA adjuvant, which basically targets TLR3 and helps in the activation of the DC population owing to this receptor. The adjuvant used in this study not only broadens the pattern recognition receptor (PRR) target range but also is a promising alternative to toxoid-based adjuvants for oral vaccination (153). In our opinion, this advancement in adjuvant discovery for mucosal immunity can offer a new hope for combating pathogenic strains and can achieve a successful vaccine design.

## The current status of adjuvants and clinical trials

Safety has always been a great concern in the process of developing novel vaccine adjuvants; therefore, many adjuvants have been comprehensively evaluated in both preclinical and clinical studies. A very commonly used adjuvant, e.g., alum, has been approved in the United States. Alum-adjuvanted vaccines were approved more than 70 years ago. Influenza vaccine in formulation with the emulsion-based vaccine adjuvant MF59 was permitted in 1997 and marketed in Europe. In addition, an emulsion-based vaccine adjuvant such as AS03 was adjuvanted with the influenza vaccine and licensed in 2009. Virosomes, a liposomal adjuvant, were approved in 2000 as an essential part of hepatitis A and influenza vaccines. In addition to this, AS04, a combination adjuvant containing MPLA adsorbed to alum, has been approved in Europe and licensed in the United States. Adjuvants that are licensed for use in humans are listed in **Table 1**.

**Table 1:** Licensed vaccine adjuvants for human use.

Adjuvant name	Formulation type	Licensed (Year)	Description	Disease type	Vaccine Trade Name
**Alum**	Aluminum as mineral salt	1962	Insoluble particulates of hydroxide, phosphate, or hydroxyphosphate sulfate salts. Adsorption of antigens by salt; modulates humoral immunity; Th2 type of immune response; increases inflammation	Diphtheria, tetanus, pertussis, inactivated poliomyelitis vaccine, hepatitis A and B, human papilloma virus, meningococcal, and pneumococcal	Daptacel, Twinrix, Gardasil, Bexsero, Prevnar 20
**MF59**	Oil-in-water emulsion	1997	Enhances recruitment of APCs and their activation; promotes antigen uptake and migration of immune cells to lymph nodes; modulates humoral and cellular immune responses	Influenza (both pandemic and seasonal)	Fluad
**AS03**	Oil-in-water emulsion	2009	Induces the production of cytokines and recruitment of immune cells. Modulates humoral and cellular immunity.	Influenza (pandemic) Used in influenza vaccine during the 2009 H1N1 pandemic	aTIV, Pandemrix, and Arepanrix
**Virosome**	Liposome	2000 These are no longer in use	Promotes uptake of vaccine antigen by APCs and interacts with B cells leading to T-cell activation. Modulates humoral and cellular immune responses.	Hepatitis A and influenza	Epaxal
**AS04**	Alum-adsorbed TLR4 agonist	2005	Stimulates TLR-4, increasing APC maturation, and imparts the Th1 type of immune responses. Improves humoral and cellular immune responses.	Human papilloma virus and hepatitis B virus	Cervarix, Fendrix
**RC-529**	Synthetic TLR4 ligand adsorbed to aluminum hydroxide	2004	Induces signal through TLR4, resulting in the upregulation of cell-surface costimulatory molecules and receptors, cytokines, and chemokines	Licensed product in Argentina for hepatitis B virus	Supervax
**Imiquimod**	Synthetic TLR7 agonist	1997	It activates Langerhans cells, which, in turn, travel to lymph nodes and induce T-cell response	Genital and perianal warts, actinic keratosis	Aldara
**Alhydroxiquim-II**	Alum adsorbed to TLR7/8 agonist	2022	Small molecules of Alhydroxiquim-II travels to lymph nodes and detaches from alum to activate two cellular receptors TLR7/8	COVID-19	COVAXIN
**CpG ODN (1018 ISS)**	Soluble TLR9 ligand (oligonucleotide) co-administered with HBV vaccine	2012	Boosts the humoral immune response, Th1-type immunity, CD8+ T-cell-mediated immunity	Hepatitis B	Heplisav-B
**CpG ODN (1018 ISS)**	Soluble TLR9 ligand (oligonucleotide) adsorbed to alum	2022	Increased cellular and humoral immunity with significant Th1-specific cytokine expression	COVID-19	CorbeVax

Despite offering many noteworthy benefits such as reduced cost, high yield, and better safety of highly purified vaccine antigens, most of these provide a weaker vaccine potential due to the lack of intrinsic immunostimulatory factors like various PAMPs. It is essential to understand how immune cell receptors such as TLRs are stimulated by PAMPs, i.e., LPS of Gram-negative bacteria, and how the adaptive immune response is coordinated and regulated by the innate immune response (154). It has been reported that particulate material stimulates innate immune signals (155). Thus, adjuvants were identified for stimulating the specific receptors on innate immune cells that can easily sense danger signals and cellular stress.

Unfortunately, due to the high reactogenicity and poor tolerance, the early developed adjuvants could not reach clinical development. Because of the characterizations of HIV and malaria vaccines, the rationale that a combination of different adjuvants is a better option to synergistically promote a broad range of immune responses, i.e., humoral and cellular, emerged (80, 156, 157). This combination of adjuvants involved a delivery system, i.e., liposomes, alum, or emulsions, as well as a natural immune stimulator immune potentiator such as bacterial MPLA, viral dsRNA, or the plant Quillaja saponins. The new-generation adjuvants are mostly known as immune stimulators or immune potentiators, which have been tested in humans and are under clinical trials listed in **Table 2**. These new-generation adjuvant systems were designed based on existing drug delivery models and established pharmaceutical principles (170). Thus, the delivery system performed is mainly on immune signal activation that could be localized and focused on the formulated vaccine antigen (54, 76, 171–174). The adjuvant system (AS) of GSK is the best example of this advancement to make an effective vaccine against malaria (80). How important is the delivery system for vaccine development? It was mentioned and emphasized in the human clinical trial of two alternative vaccines of GSK. Both vaccines contain the same immune potentiators, but they were formulated differently. The vaccine that contained the AS01 adjuvant system (a liposome formulation) provided better protection in comparison to the vaccine containing the same constituents in the AS02 adjuvant system (an emulsion formulation) (158, 168). Therefore, the vaccine candidate RTS,S was formulated with the adjuvant system AS01, and this example proved that the combination of two immunopotentiators is necessary to achieve the goal. The adjuvant system AS01 contains both MPLA and the saponin QS-21. Studies suggest that when these two immune potentiators (MPLA and QS-21) were formulated in liposomes, they work synergistically and evoke an innate immune response that further augments the adaptive immune response (158, 168, 174–176). The capacity of the AS01 adjuvant system to stimulate the robust T-cell immune response in humans for various vaccine antigens is elucidated by the synergistic effect imparted by this unique combination of immunopotentiators, e.g., MPLA and QS-21. These findings open the doors and provide new avenues for the development of potential adjuvant systems (177).

**Table 2:** Vaccine adjuvants in the process of development.

Adjuvant name	Formulation type	Description	Clinical development stage	Disease name/vaccine	References
**AS01**	Liposomes, dispersed lipid vesicles containing TLR4 ligand (MPLA) and saponin QS-21	Augments the antibody titer, Th1 type of immune response, and CD8+ T-cell-mediated immunity	Phase III	Malaria, (RTS,S) and for approval for herpes zoster vaccine (HZ/su)	(158)
**ALF**	Liposomes containing saturated phospholipids, cholesterol, and monophosphoryl lipid A (MPLA)	Imparts adequate humoral and cellular immune response	Phase I	Malaria (FMP013, FMP014), HIV-1 (gp140)	(82)
**Topical cream with TLR7 ligand**	A topical cream of TLR7 ligand (imiquimod) applied in conjunction with intradermal immunization	Promotes the innate immunity	Phase III	Influenza	(159)
**EGVac system**	Bacterial polysaccharide/bacterial DNA	Augments the immune response against HPV *via* both B-cell (humoral) and T-cell (cellular) immune signal stimulation	Phase II	Human papilloma virus	(160)
**Saponin complexes (ISCOM, Matrix-M)**	Lipid, purified saponins, and cholesterol cage-like nanocomplexes	Augments the antibody titer, and Th1 and Th2 type of immune response including CD8+ T-cell-mediated immunity	Phase I	Influenza	(161)
**GLA-SE Glucopyranosyl Lipid A (GLA)**	Oil-in-water nano-emulsion (SE) with synthetic TLR4 ligand	Multifunctional immunomodulatory activity including the production of inflammatory cytokines, chemokines, DC maturation, and antigen-presenting functions	Phase II	Tuberculosis, RSV, and Leishmania	(162)
**IC31**	Cationic peptide complexed with TLR9 ligand (oligonucleotide)	Modulates a robust H4-specific IFN-g response	Phase I	Tuberculosis	(163)
**Water-in-oil emulsions (ISA51)**	Oil dispersed nano-emulsion (mainly squalene) stabilized with non-ionic surfactant	Augments antibody production and significant Tc cell activity	Phase II	Included in licensed seasonal influenza	(164)
**VAX2012Q, VAX125**	TLR5 ligand protein (flagellin) linked to antigen	Augments the antibody titer including Th1/Th2 type of immunity	Phase II	Influenza	(165)
**Poly I:C (Ampligen, rintatolimod) PIKA**	Double-stranded RNA polymer analogue and TLR3 ligand	Augments the antibody titer and Th1 type of immune response including CD8+ T-cell-mediated immunity	Phase II	Influenza and rabies	(166)
**VCL-HB01 (Vaxfectin)**	Cationic liposome (prophylactic and therapeutic, and DNA based)	Augments T cells and antibodies	Phase II	Genital herpes	(167)
**AS02**	Oil-in-water nano-emulsion with TLR4 ligand (MPLA) and saponin, QS-21	Augments the antibody titer and Th1 type of immune response	Phase II (withdrawn)	Malaria and HIV	(168)
**Matrix-M**	Protein-based nanoparticle vaccine technology	High neutralizing antibody titer and also induces a robust T-cell immune response	Phase II	COVID-19	(169)

The rationale of most of the adjuvants as a candidate or as a licensed vaccine (54, 76, 171–174, 178–187) became clear from the data collected from clinical studies (69, 188, 189). Typically, these adjuvants are known to stimulate the innate immune response very quickly and promptly at the immunization site, draining the stimulated immune cells into the lymph nodes, which is essential to augment the pathogen-specific adaptive immunity. The stimulation of PAMP and danger signal pathways, i.e., TLR, caspase-1, and lysosomal destabilization-Syk/Card9, promotes not only the recruitment of effector cells, including T and B cells, but also the recruitment and activation of APCs. It has been proven from many studies by targeted depletion of the activated APCs that this step is very important to induce vaccine antigen-specific T cells effectively. The type of antigen-specific T-cell phenotypes that will be produced will depend on the nature of innate immune signal activation. The secretion of IL-18 by stimulated subcapsular macrophages may promote the development of IFN-γ-secreting CD4+ T cells (178, 190). Similarly, the secretion of IL-6 or IL-12 by stimulated APCs may also promote the production of a follicular-helper phenotype of T cells (TFH), which promotes the secretion of high-avidity IgG by B cells, for example, influenza immunization (191). The adjuvant system that contained TLR ligands can modulate the clonal arrangement and quality of the repertoire of antigen-specific T cells by stimulating the expansion of clones of T cells with better TCR affinity (40, 192).

Novavax, Inc. has developed an important adjuvant named matrix-M. It is based on a saponin compound derived from the bark of *Quillaja saponaria* (Soapbark tree). Currently, the matrix-M adjuvant is being used in formulation with the recombinant spike (S) protein against SARS-CoV-2. At present, this vaccine formulation (COVID-19 vaccine) is in Phase I clinical trial. The matrix-M adjuvant not only induces the production of high neutralizing antibody titers but also induces a robust T-cell immune response and imparts high protective efficacy against various strains of coronavirus (193–195). Matrix-M has also been used in other vaccine formulations such as malaria vaccine (R21/Matrix-M), which is currently in Phase IIb, and influenza vaccine, which reached Phase I (196, 197). It has been observed that saponins such as Q-21 possess adjuvant activity by inducing an OVA-specific CTL response and high antibody titer by activating caspase 1 in subcapsular sinus macrophages (SSMs) in the draining lymph node (178, 198, 199), by activating tyrosine-protein kinase SYK through destabilizing lysosome upon endocytosis (181). In addition, saponins such as matrix-M play a crucial role in the activation of the innate immune system, allow for dose-sparing in vaccine usage, and induce both humoral and cell-mediated immune responses. However, the molecular mechanism of this adjuvant remains to be elucidated. The mechanism of action of this adjuvant can be defined by using a system vaccinology approach (32).

At present, the mechanistic approach and the rationale are correlated with the effectiveness and the acceptability of adjuvanted vaccines in humans. This piece of information might be helpful in providing a protocol on how to assess and characterize new adjuvant systems for vis-to-vis comparisons in nonclinical models (200–204) and in the clinic as well (69, 103). A recent study has shown that five different adjuvanted vaccines only affect the quantities of CD4+ T cells specific to vaccine antigen but failed to modulate the range of phenotypes of these cells (103). Hence, it is not always true that adjuvants affect the quality of the immune response for all antigens. In general, vaccine effectiveness is also heavily reliant on the vaccine antigen itself and on what kind of immunity is needed for protection, for example, HIV vaccine studies (204–206).

## Use of adjuvants during the COVID-19 pandemic and future perspective

To date, we have achieved many insights into a new generation of adjuvants in terms of their immunology, and these insights may be quite helpful in providing future avenues to develop novel adjuvanted vaccines by selecting the suitable combination of adjuvants and antigens (207–209). With the advent of omics and system biology, one can easily elucidate the complicated human immune response stimulated by the ideal vaccines. Through these studies, a unique collection of molecular signatures can be compared with newly characterized vaccines (206, 209, 210). Moreover, compatible animal models can also be developed and advanced for the evaluation of potential adjuvanted vaccines. In addition, in the clinic, most of the molecular analyses are done based on peripheral blood samples and nonhuman primate models (203), which allows the evaluation of local immune response to immunization and provides the chance for logical validation of already available peripheral blood signatures. For the speedy development of new-generation adjuvants, nonclinical studies always play a crucial role in bridging clinical interpretations.

Adjuvants consist mostly of natural components. Current technologies are continuously evolving for the development of new adjuvants; soon, there will be important advancements for adjuvants containing synthetic components. While the approved adjuvants will always be considered valuable assets for the future, the key components of many of these adjuvants have been developed from natural resources and could be exchanged by synthetic molecules that possess a similar function. New molecules that have the potential to become more effective activators and agonists are currently characterized. These molecules can be efficiently produced in bulk at a low cost as they can be easily purified and evaluated and less susceptible to their intrinsic biological variability. In addition, the process for the large-scale production of these synthetic molecules may be less susceptible to the many difficulties and challenges of sourcing and extracting natural components. However, before replacing the natural components in existing adjuvants, the concern about safety and protective efficacy should be addressed in clinical trials for the vaccine in formulation with these new adjuvant candidates. As previously discussed, several promising adjuvant candidates have been studied over the past 100 years; however, only a few adjuvants were approved for human use. The complex acylated polysaccharide emulsan produced by a bacterium, *Acinetobacter calcoaceticus*, can be a good candidate due to its amenability to structural tailoring and its emulsification behavior. We found that emulsan activates macrophages and showed significant adjuvant activity as determined by hapten-specific antibody titers. This immune response was characterized by a high immunoglobulin G2a titer, consistent with a Th1 response. The significant immune potentiation demonstrated by this complex polymer establishes emulsan as an exciting new candidate adjuvant. We proposed that by manipulating the emulsan chemical structure, we can explore the physical basis of PRRs and macrophage activation (211).

In the last few years, we gained a deep understanding and immense knowledge of innate immune signals that provide new avenues to focus on adjuvant targets with higher potentials, i.e., TLR, stimulator of interferon genes (STING), retinoic acid-inducible gene I (RIG-I), C-type lectin receptors (CLRs), nucleotide-binding oligomerization domain, leucine-rich repeat and pyrin domain-containing (NLRP), and interferon-inducible protein (AIM2). In addition, some complex exploratory adjuvant concepts have been developed, including multimeric formulations containing many distinct components (212). Despite having encouraging results in preclinical studies, such adjuvants hardly play any role in ideal prophylactic vaccines, but with the advent of therapeutic vaccines, there may be additional avenues, if bulk production, robustness, reproducibility problems, etc., can be resolved. Despite having a huge advancement in adjuvants, there are some noteworthy limitations such as the incapability to stimulate a strong CTL response in humans. However, to stimulate a broad and diverse immune response, the adjuvants may also be utilized in prime/boost backgrounds with the inclusion of nucleic acids and vectors (213). Such an attempt has been made to develop an effective vaccine against HIV using the prime/boost technique, and an adjuvanted vaccine antigen is being evaluated as a booster protein after priming with a vector (214). Therefore, clinical studies are extremely essential to define the best combinations of a heterologous prime/boost setting.

Several basic problems can be solved by using a better approach to characterizing the newly developed adjuvants, i.e., by elucidating the signaling cascades specific to the innate immunity, which is required to support the potentiality of a specific vaccine. How is the impact of a quick and prompt stimulation specific to vaccine efficacy? Is there any redundancy among the signal pathways of innate immune response? Is it possible to provide a better result through a more specific stimulation? What are the exact temporal and spatial associations between the effectors of innate immunity such as cell–cell interaction and cytokine signaling, and the adaptive immune effectors such as B and T cells? Are these reactions time-dependent, in which vaccine antigens and adjuvants remain at the vaccination site, and how are the local APCs activated? In human populations, how are the adjuvants’ specific immune responses affected by ecological factors and individual genetic predispositions? What about the influence of several concurrent medications and already diseased individuals on immunization?

## Conclusion

The ultimate objective of immunization is to provide effective and long-lasting protection against various infectious diseases. Such kind of immune protection can only be achieved by using vaccine formulations carrying suitable adjuvants and antigens. Adjuvants are essential components of vaccine formulations and can modulate the vaccine response. Earlier methods of vaccine formulations focused on a single type of adjuvant, i.e., alum or emulsion. However, the new vaccine formulations need to stimulate the well-defined cellular and humoral immune responses. Therefore, to trigger robust immune responses, such as humoral and cellular immune responses, there is an utmost need for new immune potentiators or immune stimulant adjuvants in vaccine formulations. The mucosal adjuvant dmLT is the outcome of 25 years of research. It has been proven by both preclinical and clinical studies that dmLT is a safe and potent adjuvant that can induce protective immunity in a suitable formulation. There is a need to focus on some unanswered questions for the improvement of the dmLT adjuvant such as safety and stability issues of antigen–adjuvant combinations. The mechanism of adjuvanticity and the induction of protective immunity imparted by dmLT also warrant further studies. It has been suggested that dmLT can promote both innate and adaptive immune responses in the case of infants. Vaccine preparation with dmLT has been found to be effective against mucosal infections. It has been concluded that dmLT will be useful to overcome hypo-responsiveness to other whole cell- or LPS-based vaccines such as cholera, *Salmonella typhi*, and *Shigella* vaccines in infants in economically less developed countries (215). We predict that the use of dmLT in vaccine preparations for infants will be highly effective in the future.

In the recent past, many insights into the area of adjuvant research have been obtained; now, there is a choice to select a suitable adjuvant rather than a classical adjuvant, such as immune potentiators or combinations thereof, to augment vaccine efficacy. Recently, alum, MF59, AS03, CpG, and matrix-M have been licensed and used in vaccine preparations worldwide against COVID-19 (216). It has been suggested that synthetic gene coding for the spike protein, whether prefusion stabilized or also receptor binding domain only, is used to engineer mammalian cells, baculovirus, or plant cells to produce the recombinant protein that then is purified, combined with adjuvants, and used as vaccine. During vaccine preparations against COVID-19, we have gained industrial and clinical knowledge with protein-based vaccines combined with licensed adjuvants, and we believe that, in the future, vaccine preparation based on these methods will be well tolerated, effective, and available in large quantities. Furthermore, the use of maturation of RNA and viral vectors to create vaccines during the COVID-19 pandemic has opened up new avenues to produce more effective vaccines in the future for emerging infections. Recently completed clinical studies with new adjuvants recommend that a panel of new immune potentiators or immune stimulators be used in vaccine formulations for human use in the future. The accessibility of these new-generation adjuvants in different combinations will enable rational strategies for the development of successful vaccines.

## Author contributions

SV, AJ, and RR have initiated and conceptualized this review. MS was written by SV, AJ, PM, and RA. Figures and tables were created by NS, PM, and AG. Revisions were made by RA, PM, AJ, RR and SV. All the authors have designed and recommended the contents and approved the final version.
